# Real-Time Moving Object Tracking on Smartphone Using Cradle Head Servo Motor

**DOI:** 10.3390/s24041265

**Published:** 2024-02-16

**Authors:** Neunggyu Han, Sun Joo Ryu, Yunyoung Nam

**Affiliations:** 1 Department of ICT Convergence, Soonchunhyang University, Asan 31538, Republic of Korea; az0422sch@sch.ac.kr; 2 Department of Enterprise School, Soonchunhyang University, Asan 31538, Republic of Korea; 3 Department of Computer Science and Engineering, Soonchunhyang University, Asan 31538, Republic of Korea

**Keywords:** moving object tracking, cradle, head servo motor, real-time tracking, smartphone

## Abstract

The increasing demand for artificially intelligent smartphone cradles has prompted the need for real-time moving object detection. Real-time moving object tracking requires the development of algorithms for instant tracking analysis without delays. In particular, developing a system for smartphones should consider different operating systems and software development environments. Issues in current real-time moving object tracking systems arise when small and large objects coexist, causing the algorithm to prioritize larger objects or struggle with consistent tracking across varying scales. Fast object motion further complicates accurate tracking and leads to potential errors and misidentification. To address these issues, we propose a deep learning-based real-time moving object tracking system which provides an accuracy priority mode and a speed priority mode. The accuracy priority mode achieves a balance between the high accuracy and speed required in the smartphone environment. The speed priority mode optimizes the speed of inference to track fast-moving objects. The accuracy priority mode incorporates CSPNet with ResNet to maintain high accuracy, whereas the speed priority mode simplifies the complexity of the convolutional layer while maintaining accuracy. In our experiments, we evaluated both modes in terms of accuracy and speed.

## 1. Introduction

With the continuous improvement in smartphone performance and widespread use of deep learning technology, object detection techniques based on smartphones using a cradle servomotor have gained research attention [1,2,3]. Real-time moving object detection technology has been widely used in various fields, such as robotics, transportation, manufacturing, and security. With the continuous growth of the creator economy, the total addressable market is projected to reach USD 480 billion by 2027 [4]. With the increasing number of YouTube creators and the size of the YouTube market, the demand for advanced technology to support content creation is also increasing. YouTube creators rely mainly on artificially intelligent smartphone cradles for content creation. Real-time moving object detection technology has undergone rapid development owing to its inherent portability and user friendliness.

Hwang and Liao [1] developed a system that used a moving camera with a servo cradle to enable a robot to track and imitate human motions. The system achieved real-time pose imitation of 3D human motions by separating and tracking the upper and lower body movements and then recombining them during the tracking process. A servo cradle head RGB-D vision system (SCH-RGB-D-VS) was used to capture the 3D motions of the target human (TH) for imitation [1]. Servomotors controlled the movement and orientation of the camera or sensors, ensuring that the TH motions were captured correctly within the field of view (FOV) of the vision system. This allowed the humanoid robot to imitate the poses and movements of the TH in real time. 

Asali et el. [2] presented a real-time video-tracking system for visual servo applications using support vector machines. The system aimed to maintain the position of a target object in the FOV of the camera by tracking it in complex environments and lighting conditions. It combined a Sony FCB-EV7520 camera and the STRUCK algorithm for tracking, whereas a proportional–integral–derivative controller was used for visual servo control. The experimental results demonstrated the effectiveness of the system in the real-time tracking of moving objects in indoor and outdoor settings. 

Shu et al. presented an automatic camera servo system using an improved frame differential algorithm to detect and track moving objects in real time [3]. The system analyzed video streams, set appropriate thresholds to distinguish noise from movement, and sent control orders to the cradle head via a serial port for tracking. It performed well under various circumstances and incorporated video capture, temporal matching, video preservation, compression, and auto-alarm functions for monitoring.

However, the current technology for real-time moving object detection systems in smartphones faces several limitations. If small and large objects are present simultaneously, the algorithm may prioritize larger objects or struggle to maintain consistent tracking across objects of varying scales. It is difficult to accurately detect and track objects when they are occluded or overlapping. In scenarios where multiple objects are close together or obstructed, the algorithm stops tracking or tends to track only new objects appearing later in the smartphone camera. The cradle performance is also affected when objects are in fast motion on a smartphone. Quick movements can make it challenging for an algorithm to accurately track objects, leading to potential tracking errors or misidentification.

In addition, the development of moving object tracking technology using cradle servos has mainly focused on computer-based research. Studying and implementing this technology in a smartphone environment presents certain differences and challenges in terms of hardware limitations and computational resource availability. Moreover, smartphone operating systems and software development have several restrictions and requirements that can add complexity to the integration of cradle servo technology. 

In this study, we designed algorithms to address object recognition, real-time object detection, and tracking using deep learning principles. This involved the application of shape, color, and motion-based classification techniques to categorize objects based on their distinctive attributes. Overcoming the challenge of tracking multiple objects, particularly in the cases of overlaps, was achieved by incorporating the trajectory and velocity data from classified entities. This approach guaranteed seamless and continuous tracking with a steady focus on the intended target. In addition, our algorithms exhibited the capability to discern noteworthy actions within uninterrupted motion sequences, consequently resulting in improved tracking precision.

In general, YOLO [5] is fundamentally designed for rapid object detection and classification and specializes in inferring object coordinates and classes simultaneously at high speed [6]. This paper presents the incorporation of YOLO’s advantages into smartphones to address a smartphone resource limitation and to overcome resource limitations in smartphone environments. Particularly, our approach enables the filtering of objects by class, allowing for the tracking of objects belonging to a desired class, such as humans, dogs, and cats.

In this research, two approaches are proposed for object detection. First, one of our approaches is called the accuracy priority mode, which focuses on high-accuracy detection and tracking. This mode combines CSPNet [7], which employs residual blocks, with ResNet [8], a structure that divides the input into two branches. One branch passes through the network, whereas the other is concatenated with the resultant features. The other approach is called the speed priority mode, which prioritizes a fast inference rate. This mode is specifically designed for rapid processing and achieves notable speed improvement. The speed priority mode reduces the number of layers while maintaining a reasonable level of accuracy. Within a single application, users can choose between the two approaches based on their priorities. The first approach enables users to prioritize accuracy, making it suitable for tasks such as accurate differentiation between dogs and cats. However, in scenarios involving rapidly moving objects, the first approach may prove inadequate, rendering the second approach a more suitable choice. 

The object-tracking process involves a cradle servo that continuously tracks objects and accurately positions them at the center of a camera. This is achieved by calculating the object velocity through an assessment of positional changes between consecutive frames, as well as by measuring the spatial distance between the object and the focal center of the camera.

Furthermore, when tracking objects, instead of maintaining a constant speed, the algorithm is designed to track objects at variable speeds based on the coordinates of objects. Unlike fixed-speed tracking, which may miss objects or result in unnecessary cradle movements, our research compensates for these drawbacks by enabling tracking at speeds matching the object’s movement. Inspired by the fact that objects moving faster may quickly exit the screen, we adjusted the cradle’s rotation speed to be faster towards the edges of the screen and slower towards the center. This approach ensures continuous tracking without missing objects.

The implementation of the two modes and cradle servo systems involved the following research processes: Initially, a dataset consisting of 100 YouTube videos was collected, from which specific data related to humans, dogs, and cats were extracted, resulting in a dataset of 65,000 frames. Following data extraction, pre-trained weights were applied for preliminary labeling and review. Subsequently, an accuracy priority mode was developed with a primary focus on achieving high accuracy while minimizing computation costs. The mean average precision at 50 Intersection over Union (mAP50 IoU) was adopted as the accuracy evaluation metric for the performance assessment. Moreover, the computational cost of the mode was quantified using giga-floating-point operations. The mode architecture was established by integrating elements from the ResNet structure introduced in YOLOv3 [9] and CSPResNet utilized in YOLOv4 [10]. A comprehensive evaluation of computational cost and accuracy was conducted to determine the optimal model. Finally, the derived model served as the basis for developing a speed priority variant by simplifying the number of layers by testing multiple times to determine the optimal number of layers to fit into our model.

To summarize, our contributions are as follows:

- We propose a modified CSPNet structure to overcome computational resource limitations and improve overall system performance in smartphone environments for object detection. We improve recognition and classification accuracy and significant inference speed compared to other configurations of moving detection methods.
- We improve the performance of seamless object tracking by incorporating the dynamically changing speed of objects. This integration minimizes unnecessary tremors and disruptions in tracking, achieved through the perspective projection of a camera model. Consequently, it enables the effective regulation of the cradle motor, facilitating optimal tracking mechanisms.
- Our experimental results show our approach outperforms other related methods. The results were compared with both accuracy-centric and speed-centric modes for real-time object detection and tracking applications.

## 2. Materials and Methods

### 2.1. Proposed Methodology

**Figure 1:**
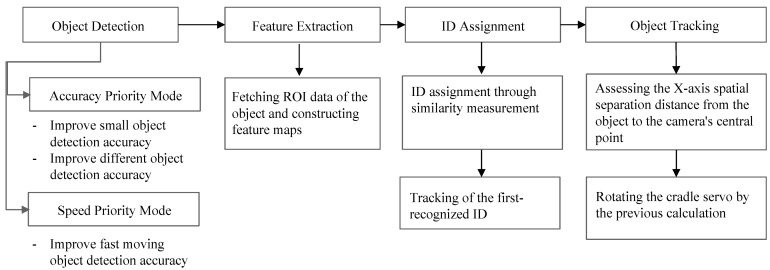
Flow diagram of the proposed method.

Figure 1 shows a diagram of the proposed system. This system is designed to track moving objects in real time based on videos captured using a smartphone cradle servo. The first object-tracking process involves the detection and recognition of objects. To mitigate the existing issue of objects, particularly small ones, not being recognized from a distance by the camera, an accuracy priority mode was developed to ensure the recognition of small objects. We aimed to enhance the accuracy of classification to effectively distinguish between objects such as humans, dogs, and cats, while simultaneously improving the accuracy of recognition, regardless of the size of the object and its distance from the camera.

Furthermore, to enhance the tracking accuracy of rapidly moving objects, we developed a speed priority mode with a focus on optimizing processing speed. The conventional YOLO model is designed to excel on the COCO dataset [11], which comprises 80 classes and presents limitations in terms of a lightweight model. The COCO dataset requires the precise classification and recognition of a wide array of everyday objects across 80 classes. Even the latest version, YOLOv8, could only achieve an accuracy of 55% in terms of mAP50-95 evaluation. However, this study primarily aimed to refine the servo usage of content creators. Given that prominent objects mainly include humans, dogs, and cats, this study targeted the recognition of these entities. Consequently, because only three classes must be classified and recognized, there is potential for a more lightweight and streamlined approach. 

The aforementioned accuracy and speed priority modes were developed to be selectable by users, allowing mode changes as required. Both the accuracy and speed priority modes were grounded in enhancing the recognition speed in the smartphone environment. Due to limited resources of smartphones, it is necessary to develop lightweight detection models to ensure real-time tracking. In addition, the extracted feature maps of the detected objects were compared for similarity in order to assign IDs. During the extraction of the object feature maps, data from the region of interest of the recognized object were obtained. These data were passed through a convolutional neural network (CNN)-based feature extractor and transformed into feature maps. The CNN-based feature extractor comprised CNN layers trained on humans, dogs, and cats. In the ID assignment process based on feature maps, the features of the object in the current frame were compared with those of the previous frame. If the similarity between existing and current feature maps exceeded 50%, the same ID was reassigned. Otherwise, a new ID was assigned.

Subsequently, the design of the smartphone cradle servo was tailored to follow the identification tag of the foremost-recognized object within a pool of multiple detected IDs. This engineering approach aims to mitigate the tracking inaccuracies stemming from the simultaneous presence of objects with various sizes. The primary users of the cradle servo are predominantly content creators and, in most instances, the earliest-appearing object assumes the role of the main subject. Consequently, the servo design prioritizes a tracking paradigm rooted in the order of recognition, independent of the object dimensions, with this particular use-case taking precedence. Although the possibility of selectively tracking a designated object in isolation is viable for experimental purposes, a simplified iteration was incorporated.

Moreover, the position of the tracked object was aligned with the central axis of the camera. This alignment was established through a process involving the computation of the measurement of spatial separation from the focal center of the camera. The methodology employed the strategic adjustment of the coordinates of the object, facilitating its relocation to the exact center of the display of the camera. This procedural approach ensured the consistent maintenance of the central alignment of the object with respect to the viewpoint of the camera. Figure 1 shows the flow diagram of the proposed method.

### 2.2. Experimental Environment

The experimental setup consists of two distinct environments. The training environment is presented in Table 1, and the implementation environment for the smartphone is presented in Table 2. The environmental setup for training the YOLO model involved Docker, wherein the nvidia/cuda:11.6.2-cudnn8-devel-ubuntu20.04 image was selected from a range of Docker images. This specific image encompassed NVIDIA’s CUDA Toolkit and cuDNN. This environment was used as the backdrop to augment the capabilities of the existing YOLOv8 platform. This augmentation was achieved through adjustments tailored to the construction of desired models. The original platform lacked specific essential layers, such as “Shortcut”, pivotal for configuring novel model architectures. Hence, resources were applied to a modified platform that featured the requisite additional layers, thus facilitating model training.

Android 13 was used as the operating system during the experimental stage. YOLOv8 was used for object recognition and tracking algorithms. YOLOv8 uses an innovative repository to support object detection and instance segmentation [12]. This provides an integrated weight converter, enhancing portability across various libraries. Its architectural structure is characterized by a relatively shallow design, differentiating it from alternative versions and enabling uncomplicated customization. YOLOv8 employs an anchor-free model that directly anticipates the center of the object, diverging from the use of anchor box offsets [13]. Consequently, this approach accelerates the non-maximum suppression process.

TensorFlow Lite was used as the inference framework to implement the application. YOLOv8 is typically implemented using Torch. Torch-based smartphone frameworks have a performance bottleneck owing to the lack of GPU support. TensorFlow Lite, however, allows easy GPU acceleration and provides example code, making it a more efficient choice for mobile applications. YOLOv8 was converted using TensorFlow Lite instead of Torch. 

YouTube video data were utilized as the primary dataset owing to their availability and diverse conditions, including composition, angle, and brightness. A total of 100 videos were collected to ensure an extensive and representative dataset encompassing various resolutions ranging from HD (1280 × 720) to UHD (3840 × 2160). Because the YOLOv8 platform primarily uses images for training rather than videos, frame data were extracted from the collected videos at a rate of two frames per second (fps) to create image datasets for training. The extracted frames were subjected to a labeling process before being used for training. 

The labeling process involved automated labeling using pretrained weights, followed by a validation stage. Automated labeling employed the pretrained weights of YOLOv8x, which were divided into inference and post-processing stages. During the inference stage, the pretrained weights of YOLOv8x were employed to infer the results for the images in the dataset. Given that YOLOv8x was trained on 80 classes, the results were obtained for all 80 classes. From these results, the process of interest involved selecting and computing the coordinate values for the desired classes, which then formed the basis for labeling, constituting the post-processing stage. In this post-processing phase, all data except for data involving humans, dogs, and cats were discarded, and the subsequent steps involved the computation of the central coordinates along the x- and y-axes, as well as the box dimensions. This culminated in the generation of the label data.

Following the automated labeling process, a manual validation step was performed on each label dataset. During this phase, corrective actions were taken when erroneous object labeling or incorrect class assignments occurred, such as cases in which ordinary garments were erroneously labeled as humans, or dogs were mistakenly labeled as cats. In such scenarios, the respective label data were edited by either removal or class name adjustment. This comprehensive procedure was applied to a dataset of 100 videos. Upon completion of the labeling process, 90 of these video datasets were employed as the training dataset and the remaining 10 were utilized as the validation dataset.

### 2.3. Mode Configuration for Object Detection 

**Figure 2:**
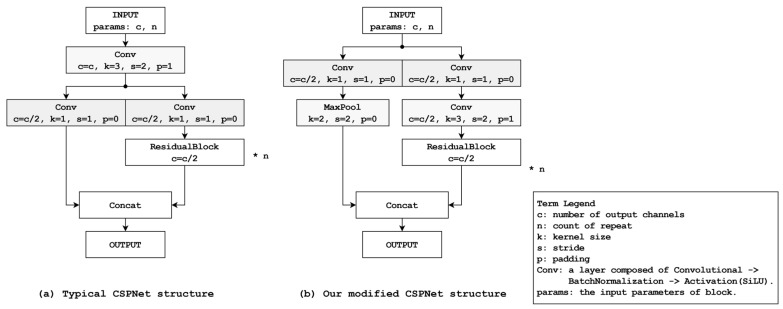
Typical CSPNet structure and modified CSPNet structure.

The object detection framework utilizes two distinct modes: accuracy priority and speed priority. The accuracy priority mode was designed to achieve better performance than the YOLOv8s model while minimizing the potential reduction in accuracy. As shown in Figure 2, the foundation is derived from a path aggregation network [14] which serves as an extension of the feature pyramid network (FPN) structure [15] deployed within the YOLOv8 context. This extension entails an additional upscaling step from the downscaled outcomes of the FPN, resulting in improved inference accuracy compared with standard FPN utilization.

In the integration of YOLOv8 into a smartphone environment, we undertook a reinterpretation of the CSPNet structure to address computational resource limitations and enhance overall system performance. Traditional implementations of CSPNet involve a doubling of feature maps extracted from images during down-sampling. However, this approach introduces computational bottlenecks, impacting processing speed significantly. Figure 2 illustrates our strategy to overcome this limitation. By preserving the number of feature maps during down-sampling, our approach achieves improved computational efficiency compared to the conventional method. It is expected to reflect this advancement, showcasing reduced FLOPs for both the accuracy priority mode and speed priority mode, thus affirming the effectiveness of our proposed approach in mitigating computational resource constraints. 

In the accuracy priority mode, a block rooted in the application of CSPNet is integrated with ResNet. This block is constructed using an adapted version of the CSPNet structure. Although CSPNet divides the input data into two segments, linking one part to the existing network and concatenating the output with the other, the specified block also segments the inputs into two divisions. One division connects the down-sampling convolutional layers and the existing network, and the other interfaces with the max-pooling layers. The outcomes of both divisions are subsequently combined. This structure combines the ResidualBlocks inspired by ResNet to form CSPResidualBlocks. Here, the residual block is configured based on the structure used in YOLOv5 [16] and YOLOv8.

In the neck segment, which serves as the intermediary between the backbone and the head, the mode commences with SPPCSP (spatial pyramid pool; SPP [16] with CSPNet) and employs ResidualBlocks instead of CSPResidualBlocks during upscaling expansion. This modification is driven by the limitation of CSPResidualBlocks within this mode to effectively accommodate the disparity between the input and output sizes. As the mode advances toward the upscaling phase, the number of filters is reduced. The CSPResNet architecture detailed in this study entails a twofold increase in the filter count relative to the input, which presents a challenge when attempting to match or halve the output size while maintaining a consistent input. Such an adjustment results in an overall decrease in the number of filters across the structure, possibly leading to a loss of important features. Consequently, upon the completion of the upscaling phase, the subsequent downscaling expansion incorporates CSPResidualBlocks. During this stage, the augmented filter count facilitates the effective application of this architectural structure.

**Figure 3:**
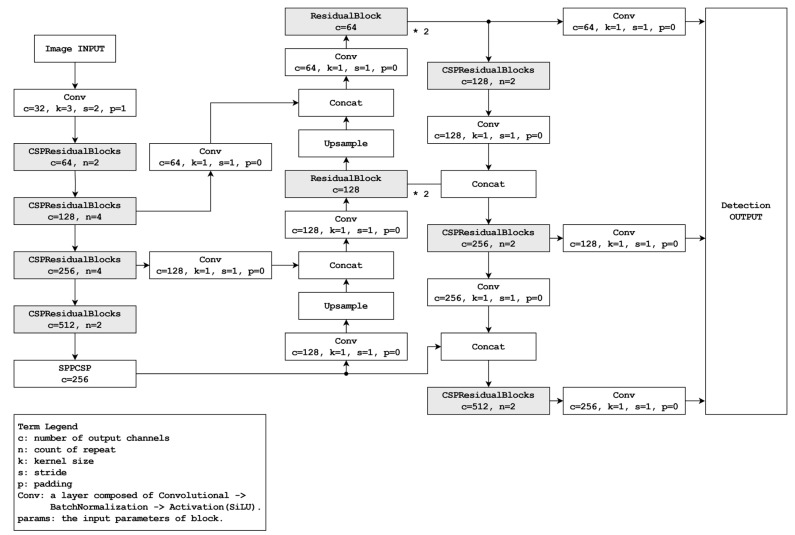
Diagram of the accuracy priority mode.

The model designed for the speed priority mode is characterized by a considerably high inference speed without significantly compromising accuracy. This particular mode streamlines its architecture by reducing the number of extraction layers and filters compared with its accuracy mode counterpart. A schematic of this mode is depicted in Figure 3.

In contrast to the accuracy priority mode illustrated in Figure 3, the current mode is characterized by only two outputs. Consequently, the output shape is simplified while maintaining a consistent output size criterion. The accuracy priority mode generates an output shape of (1, 7, 8400) based on a 640 × 640-pixel input size. Conversely, the speed priority mode, under the same input size criterion, significantly reduces the coordinate count of the output array by more than four times, resulting in the shape of (1, 7, 2000). This reduction not only streamlines processing and enhances recognition speed, but also leads to reduced computational overhead.

**Figure 4:**
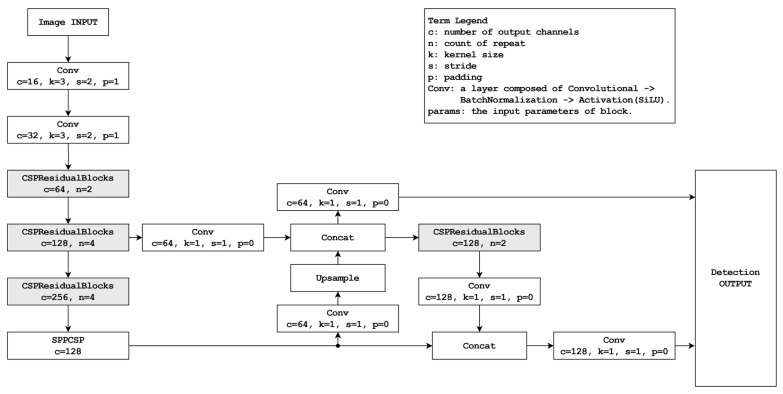
Diagram of the speed priority mode.

Regarding the backbone structure, the current model exhibits a certain level of simplification compared with the accuracy priority model depicted in Figure 4. Within this revised framework, a solitary CSPResidualBlock is substituted with a down-sampling convolutional layer. The head section is designed with increased simplicity to achieve the two desired outputs. Although residual blocks remain unused during upscaling, they are employed once during the downscaling process. This configuration facilitates the achievement of dual output.

### 2.4. ID Assigning System for Object Detection

TensorFlow Lite provides object-recognition results exclusively in the form of coordinates and their corresponding classes. However, they lack a built-in mechanism for assigning identification (ID) tags to individual object features. Consequently, implementing a custom system that efficiently allocates IDs to TensorFlow is imperative. Thus, we can effectively track and differentiate specific objects of interest. These unique IDs serve the purpose of distinguishing between various objects, such as differentiating between “Person 0” and “Person 1”.

**Figure 5:**
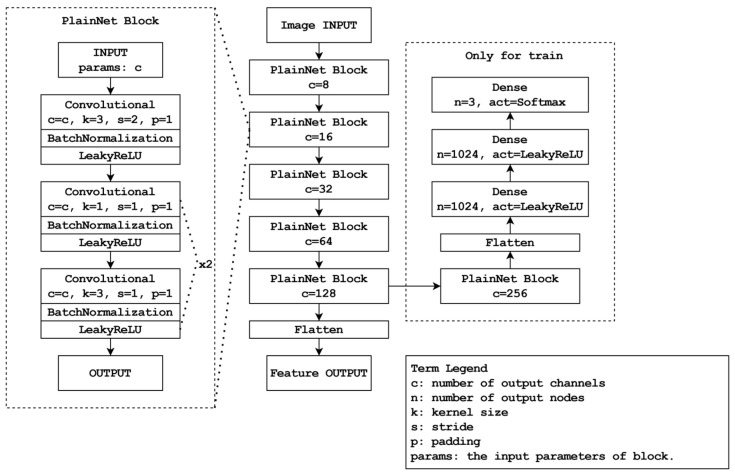
Diagram of CNN of ID assigning system.

The ID allocation system comprises two steps. Initially, feature extraction is performed using CNNs. Specifically, a CNN model called PlainNet trained on datasets encompassing people, dogs, and cats is employed to extract the features. Figure 5 illustrates the structure of the CNN model. In the next step, the system extracts features and subsequently evaluates the similarity of feature maps to either reassign existing IDs or allocate new IDs. The following equation 1 based on the cosine similarity formula is used for this purpose:

(1) $$Similarity\left(F_{prev},\;F_{curr}\right)=ReLU(\frac{\sum_{i=1}^{n}F_{{prev}_{i}}*F_{{curr}_{i}}}{\sqrt{\sum_{i=1}^{n}{F_{{prev}_{i}}}^{2}}*\sqrt{\sum_{i=1}^{n}{F_{{curr}_{i}}}^{2}}})$$

In accordance with this formula, if a feature map exhibits a similarity of 50% or higher to an already existing feature map, it is reassigned an identical ID associated with the feature map. Conversely, if the similarity is below the designated threshold, a new ID is assigned, and the corresponding feature map is associated with the newly assigned ID and subsequently stored.

### 2.5. Object Tracking 

Experimental trials were conducted to track physical objects by connecting the application to a cradle device using the API of the cradle. The API code controlling the cradle within the application was based on the code provided by Pivo. The cradle control functions included ‘turnLeft’, ‘turnRight’, ‘turnLeftContinuous’, and ‘turnRightContinuous.’ To achieve smooth and continuous movement, the ‘turnLeftContinuous’ and ‘turnRightContinuous’ functions were employed, both of which continue rotating at the speed set before the function call if no speed value is provided as an argument. The rotational speed represents the time required for the rotation to complete a 360° cycle, rather than denoting the angular velocity. Setting a rotational speed of 10 implies that the object will complete one 360° rotation in a span of 10 s. This relationship is expressed as follows:

(2) $$rt=360{}^{\circ}/v_{actual}$$

In Equation (2), the variable ‘rt’ (rotational time) denotes the time required for a complete 360° rotation, serving as an input for the cradle control function and functions as a time indicator for the cradle to complete its full rotation. Likewise, the variable $v_{actual}$ represents the real-time rotational speed of the cradle, measured in angular velocity (degrees per second). Hence, considering the characteristic that ’rt’ increases as the actual rotational speed of the cradle decreases, and decreases as it accelerates, the calculation of rotational time is facilitated.

The cradle rotation speed is defined as the duration required to complete a 360° rotation. In addition, the system is designed to offer an adjustable cradle rotation speed, enabling both rapid and gradual cradle rotation based on the proximity of the object to the central focal point of the camera. The extent of object displacement is determined by the x-axis coordinates of the object relative to the central point of the camera. Moreover, the cradle rotation duration is intentionally configured to decrease as the object approaches the center of the camera image. This feature is vital in preventing situations in which, even if the object remains stationary, the failure of the cradle to stop promptly could result in the object failing to align with the central point of the image of the camera, resulting in lateral movement. Consequently, by capitalizing on this unique attribute of the cradle, the following formula is employed to calculate the rotation time based on the distance between the object and the central point of the screen.

(3) $$MD=\left\{\begin{array}{c} 0\;(if\;0.4<OP<0.6)\\ \left(OP-0.5\right)\times 2\;(else) \end{array}\right.$$

(4) $$RT=\left\{\begin{array}{c} \frac{6}{MD}\;(if\;MD\neq 0)\\ 0\;\;\;(if\;MD=0) \end{array}\right.$$

Equation (3) describes the preprocessing steps involved in determining the x-axis central coordinates of an object. In this equation, OP (object position) signifies the x-axis coordinate of the object. It is crucial to emphasize that, for the sake of computational efficiency in this experiment, the object positions were standardized within a range of 0 to 1, measured in pixels. The distance from the screen center to the object is represented as MD (moving distance). Specifically, when the object is aligned with the center of the camera, it is designated as P = 0.5. If the object is positioned at the far-right edge of the camera, it is expressed as P = 1, and P = 0 indicates that the object is at the far-left edge of the camera. The cradle used in this study does not involve tilting, and therefore, vertical (y-axis) movement of the object is not considered.

In this equation, when the OP is in the range of 0.4 to 0.6, near the center of the screen (OP=0.5), we designed it to return MD = 0. MD represents the distance from the center of the screen to the object, as previously explained, using normalized values. Because the distance from the central point of the screen, which is 0.5, is minimal, there is no need for the cradle to move, even if the object is outside this area. For positions outside this range, we subtract 0.5 from OP and then multiply the result by 2, ensuring it falls within the range of −1 and 1. When MD approaches 1, it signifies the position of the object at the far-right edge. In such cases, the cradle must rotate more rapidly to the right to reposition the object at the center of the camera. Conversely, when MD approaches −1, it indicates the object position at the far-left edge. In such instances, the cradle moves in the opposite direction to reposition the object located at the left edge back to the center of the camera.

In Equation (4), RT (rotational time) is recalculated as the time required for the cradle to complete a 360° rotation based on the MD value. The API provided by PIVO is used in the cradle control section. The RT value spans a range from 6 to infinity. When the RT value is 6, the cradle rotates at its maximum speed, and as RT increases towards infinity, the rotation rate of the cradle decreases towards zero. The design ensures that as MD approaches 1 or -1, the RT value tends towards 6. Conversely, when the object moves closer to the center of the screen, causing MD to converge to 0, RT approaches infinity, resulting in a lower rotation speed. Furthermore, to prevent a zero-division error when the preprocessed MD value equals zero, RT is set to zero in such instances. Subsequently, the obtained RT values are used to rotate the cradle. When RT is positive, the cradle rotates to the right, and when it is negative, it rotates to the left. If RT is 0, the cradle remains stationary and no rotation occurs.

### 2.6. Visual Display Implementation

**Figure 6:**
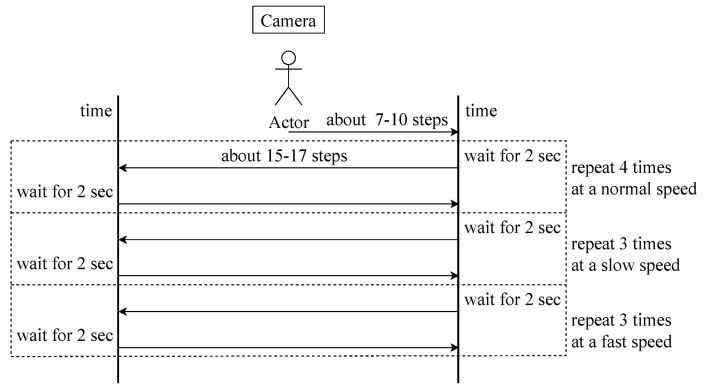
Protocol of the experiment.

**Figure 7:**
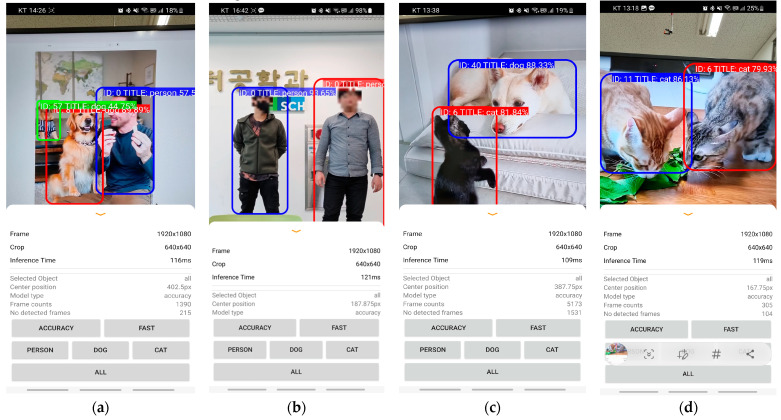
Examples of screenshots with accuracy priority mode. The different colors of each square are used to distinguish the detection order. In screenshots, blue, red, green squares are displayed in the order in which moving objects appear. (**a**) Three classes of smartphone with accuracy priority mode. (**b**) People on smartphone with accuracy priority mode. (**c**) Dog and cat on smartphone with accu-racy priority mode. (**d**) Cats on smartphone with accuracy priority mode.

Figure 6 and Figure 7 provide an overview of the object-recognition components of our visual display application. Object recognition screens have several key features. First, the two models use different input sizes. The accuracy priority mode operates with an input size of 640 × 640, and the speed priority mode uses an input size of 320 × 320. Second, users—particularly content creators—have the option of selecting between the accuracy priority mode and the speed priority mode. Third, the screen incorporates an object-selection function that enables users to specify the objects they wish to recognize. The users can choose to detect humans, dogs, cats, or other objects.

Furthermore, the screen displays an assigned ID above the recently recognized object. The ID is determined by analyzing the characteristics of the identified object, including its size, shape, and color. The assignment process ensures that the same object consistently receives the same ID, thereby facilitating object tracking over time. In this application, object selection and ID assignment features are important for the accurate and reliable recognition of objects. The application can be tailored to the specific needs of the user by allowing them to select the objects to be recognized.

### 2.7. Object Tracking Error Rate Assessment

The mean absolute error (MAE) was calculated to assess the object tracking proficiency after object recognition. MAE is a metric used to measure the average absolute difference between the predicted and actual values in a set of data [17]. This provides a simple method for assessing the accuracy of predictive models. To calculate the MAE, the absolute difference between each predicted value and its corresponding actual value is obtained, and the average of these absolute differences is computed, where n is the number of data points, *Actual_value_i_* is the actual value of the *i*-th data point, and *Predicted_value_i_* is the predicted value of the i-th data point. A lower MAE indicates a more accurate model because it represents smaller errors between the predictions and the actual data. This is expressed as follows:

(5) $$MAE=\frac{\sum_{i=1\;}^{n}\left|{Predicted\_Value}_{i}-{Actual\_Value}_{i}\right|}{n}$$

Applying the formula outlined above, the calculation of the MAE for this experiment involved determining the difference between the actual movement of the physical object and the movement of the servo cradle, divided by the value *n* and the number of data points. It is important to note that the actual movement of an object is represented as ${OP}_{i}$, indicating its movement within the camera screen rather than in the physical space. OP represents the object position on the screen in Equation (3). The movement of the servo cradle is denoted as ${OP}_{i-1}$ and, for each frame transition, it is compared with the position of the object in the previous frame. The servo cradle is designed to place the object away from the previous frame. Consequently, the coordinates from the previous frame (${OP}_{i-1}$) can be considered predicted values, whereas the coordinates from the current frame (${OP}_{i}$) can be considered actual values. This formula is expressed as follows:

(6) $$MAE=\frac{\sum_{i=2}^{n}\left\{\begin{array}{c} \left|{OP}_{i}-{OP}_{i-1}\right|\;(if\;\left|{OP}_{i}-{OP}_{i-1}\right|\geq 0.1)\\ 0\;(else) \end{array}\right.}{n-1}$$

An experiment was conducted to compare the results of object tracking using YOLOv8s with those obtained using the two methods proposed in this study. YOLOv8s was chosen because of its popularity as a widely used version of YOLOv8, known for its superior processing speed and accuracy compared with other detailed versions of YOLOv8. In this model, the object-recognition component was utilized within the context of object-tracking algorithms.

## 3. Experimental Setup and Protocol

An experiment was conducted to track objects based on their movements. The experimental procedure was as follows. A single individual was positioned in the center of the camera frame (a). Initially, the person moved approximately 7–10 steps to the right relative to the camera frame, and then stopped (b). Subsequently, they moved approximately 15–17 steps to the left and stopped (c). Finally, they moved approximately 15–17 steps to the right (d). This process was repeated approximately ten times for both (c) and (d). Ten ordinary individuals participated in these procedures, resulting in 100 experimental trials.

The tracking experiment was conducted as follows. First, a group of 10 individuals participated in the experiment. Figure 6 shows the protocol of the experiment.

1. Stand in the center of the camera frame and wait for 10 seconds.
2. Walk in the right direction from the camera frame for 7–10 steps.
3. Wait in place for 2 seconds.
4. Walk in the left direction from the camera frame for 15–17 steps.
5. Wait in place for 2 seconds.
6. Walk in the right direction from the camera frame for 15–17 steps.
7. Wait in place for 2 seconds.
8. Repeat steps 4–7 additional 9 times.

The experiment consisted of 10 rounds, where for 4 rounds, participants walked at their usual pace, for 3 rounds, participants walked at a slower pace than their usual gait, and for the remaining 3 rounds, participants walked at a faster pace than their usual gait. The time taken during walking was approximately 10 seconds for 15–17 steps in the case of the usual pace. For the slower pace, it took about 17 seconds, and for the faster pace, it took approximately 6 seconds. The time taken for the entire experiment for one participant is shown in Table 3.

## 4. Results

### 4.1. Moving Object Detection

Table 4 presents the image classification performance of CSPResNet-34 using ResNet-34, conventional CSPNet architecture, and our proposed modified CSPNet structure. The evaluation was conducted using the CIFAR-100 [16,18] dataset for training and performance comparison. For resource usage, while the amount of computations can be derived in terms of FLOPs, GPU memory usage cannot be monitored on smartphones. Therefore, monitoring was conducted in the training environment outlined in Table 1. From these results, the modified CSPResNet-34 achieves 3.35 times and 1.38 times fewer FLOPs than ResNet-34 and traditional CSPResNet-34, respectively. The inference speeds of the modified CSPResNet-34 are 2.9% and 17.6% faster for batch 1 (single image) than those of ResNet-34 and traditional CSPResNet-34, respectively. Also, the modified CSPResNet-34 uses 12.4% and 1.3% less GPU memory than ResNet-34 and traditional CSPResNet-34, respectively. Thus, our model can help overcome resource limitations in smartphone environments. 

**Figure 8:**
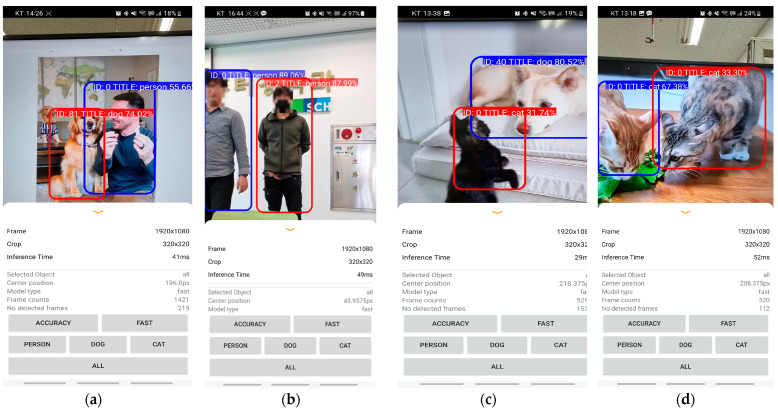
Examples of screenshots with speed priority mode. In screenshots, blue, red, green squares are displayed in the order in which moving objects appear. (**a**) Three classes of smartphone with speed priority mode. (**b**) People on smartphone with speed priority mode. (**c**) Dog and cat on smartphone with speed priority mode. (**d**) Cats on smartphone with speed priority mode.

Figure 7 and Figure 8 depict samples of research outcomes within a real-world experimental environment. This environment replicates a scenario in which physical objects are captured by a camera, and subsequent experiments are conducted accordingly. Notably, no specific measures were taken to counteract the potential performance degradation resulting from the heat build-up of the smartphone in this experiment. Additionally, the inclusion of an ID allocation system introduces additional computational resource consumption, which leads to a slightly reduced processing rate compared with the inherent capability of the model. The colors of the squares represent the detected images in the order recognized by TensorFlow Lite. The ID indicates the number of the detected object, and the Title indicates whether it recognized a person, cat, or dog. The last number indicates the confidence of the detected object. The different colors of each square are used to distinguish the detection order.

The efficacy of the proposed accuracy priority mode is substantiated by the successful recognition of two or three objects when they appear simultaneously. In addition, the model demonstrates the ability to differentiate and accurately identify multiple objects of the same type when they co-occur. Moreover, the performance analysis reveals that the speed priority mode is proficient in recognizing and tracking diverse objects, whether they are dissimilar or belong to the same category, and if they move collectively at the desired speed. However, the model exhibits certain limitations. Specifically, in instances in which more than three objects appear, the recognition results can be inconsistent, leading to successful recognition in some cases and failure in others. A summary of the results obtained from this experiment is presented in Table 5.

### 4.2. Performance Results

Table 6 and Table 7 present the comprehensive evaluation results for each mode. For a comparative analysis of the two developed modes, YOLOv3 and YOLOv8 were trained using an identical dataset. Training was conducted using the YOLOv8 platform.

In this evaluation, an accuracy assessment was performed on a validation dataset derived from our comprehensive dataset. This dataset comprised data extracted from 10 YouTube videos and encompassed approximately 4500 images. Each image within the dataset was meticulously labeled, with an average of 450 instances for the person class, 1500 instances for the dog class, and 2700 instances for the cat class.

The inference rate denotes the processing speed of the model. To accurately measure the inference rate, a controlled experiment was conducted by covering the smartphone camera and displaying a black image. This ensured that only the inference process of the mode was evaluated. The investigation highlights that the presence of recognized objects triggers a notable slowdown in the detection speed during the post-processing phase for ID allocation, resulting in a reduction of 50% or lower in the original processing speed. Furthermore, to maintain experimental fairness, the smartphone underwent sufficient cooling before conducting the measurements. This precautionary step aims to mitigate the performance degradation caused by heat, thereby ensuring the accuracy and reliability of the obtained results. 

### 4.3. Object Tracking Error Rate 

Table 8 presents the results of the object tracking using YOLOv8s and the two methods proposed in this study. When YOLOv8s is employed, the MAE of the servo cradle exceeds 0.2. However, in both the accuracy and speed priority modes, the average MAE values of the servo cradle are 0.107 and 0.103, respectively, representing a tracking distance of approximately twice that in YOLOv8s. As shown in Table 6 when utilizing an input size of 640 × 640, YOLOv8s exhibits a lower inference speed than the proposed models, resulting in a decrease in accuracy. This low inference speed hinders swift object localization and necessitates multiple lateral movements of the servo cradle. The increased frequency of the lateral movements also leads to a lower accuracy of the servo cradle. In relative terms, the speed priority mode outperforms YOLOv8s with a faster object detection, covering less than half of the MAE of YOLOv8s. The accuracy priority mode, while still falling below half the value, achieves a slightly lower accuracy than the speed priority mode.

## 5. Conclusions

The increasing demand for real-time moving object detection systems in artificially intelligent smartphone cradles has prompted the development of two approaches. The accuracy priority mode and speed priority mode were proposed to strike a balance between speed, accuracy, and inference rate. Object tracking was implemented by leveraging a cradle servo motion algorithm designed to position mobile entities at the center of the camera screen. 

The accuracy priority mode leveraged the CSPNet structure integrated with ResNet based on the YOLOv8 architecture, achieving an impressive accuracy level of 93% based on the mean average precision at 50 IoU (mAP50) metric. It employed a lower network depth and omitted bottleneck layers to enhance accuracy while maintaining a high inference rate. This mode exhibited accuracy comparable to that of YOLOv8 while demanding fewer computing resources in a smartphone environment. 

By contrast, the speed priority mode prioritized high inference rates of 50 fps and achieved a commendable accuracy rate of 90% by reducing the number of layers. Among the various YOLO models, YOLOv5n exhibited the shallowest architecture. The speed priority mode surpassed YOLOv5n in terms of computational resource requirements, while maintaining a competitive level of accuracy. Notably, the accuracy of this model was not significantly compromised compared with the other models. The combination of accuracy reduces the computational resource requirements, and high-speed performance makes these models well suited for detecting multiple moving objects in one frame in real time in a smartphone environment.

This study achieved a successful and accurate real-time recognition of objects, such as humans, dogs, and cats. However, the complexity of the CNN-based feature extractor in the ID assignment system introduced a notable drawback that led to a significant reduction in the actual inference speed. Particularly evident in the speed priority mode, the mode itself demonstrated an impressive inference speed of up to 50 fps. However, object recognition led to a drop in the frame rate to below 30 fps. Consequently, there is an urgent need to simplify the feature extractor to minimize the loss of inference speed. Furthermore, certain cases revealed scenarios in which objects were assigned different IDs owing to variations in perspectives, even when they were the same object or shared the same ID. Consequently, essential improvements, such as broadening the training classes of the feature extractor, are required to address this concern.

Finally, a practical implementation of tracking and controlling physical objects using a servomotor was achieved. This servomotor, as part of a smartphone-compatible cradle, offered free rotation capabilities spanning 360°. The cradle device, in conjunction with a dedicated API, facilitated the derivation of the necessary formulas for object tracking and enabled us to conduct experiments with real objects. The results indicated MAE values of 0.107 in the accuracy priority mode and 0.103 in the speed priority mode, significantly outperforming the YOLOv8s model with an MAE value of 0.223. This represented an approximately two-fold reduction in error compared with the YOLOv8s model without our proposed method. Hence, employing our approach can lead to enhanced tracking accuracy compared with conventional methods. However, in both the accuracy and speed priority modes, the MAE values of the servo cradle were 0.107 and 0.103, respectively, representing a tracking distance of approximately twice that of YOLOv8s.

## Figures and Tables

**Table 1:** Training configuration on a server.

Configuration	
CPU	Intel Xeon Silver 4216 x2
RAM	192 GB
GPU	RTX A5000 x2
OS	Ubuntu 20.04
YOLO Platform	Modified YOLOv8

**Table 2:** Environments implemented on a smartphone and a cradle.

Smartphone and Cradle	
Smartphone Model	GalaxyS21+
OS	Android 13
Inference Framework	TensorFlow Lite 2.8.0
Cradle Model	Pivo Pod Silver

**Table 3:** Experimental setup.

Movement Speed	Elapsed Time of Movement(s)	Waiting Time (fps)
Regular walking	80	16
Slow walking	102	12
Fast walking	36	12
Total elapsed time	218	40

**Table 4:** Performance comparison of CSPResNet-34 and modified CSPResNet-34.

Model	FLOPs (G)	Accuracy	Speed (at Batch 1)	GPU Memory Usage (at Batch 1)
ResNet-34	31.8	91.3%	3.5 ms	588 MB
Traditional CSPResNet-34	13.1	90.9%	4.0 ms	530 MB
Modified CSPResNet-34	9.5	91.4%	3.4 ms	523 MB

**Table 5:** Object detection results.

Mode	Class	Inference Rate (fps)
Accuracy Priority Mode	3 Classes	8.62
	Humans	8.26
	Dog and Cat	9.17
	Cats	8.47
Speed Priority Mode	3 Classes	24.39
	Humans	23.4
	Dog and Cat	34.48
	Cats	23.3

**Table 6:** Experimental results of models with 640 × 640 images.

Model	Input Size	FLOPs (G)	Accuracy (%)				Inference Rate (fps)
			mAP50				
			All	Person	Dog	Cat	
YOLOv3-tiny	640 × 640	**18.9**	91.5	86.3	93.3	94.9	11.7
YOLOv4-tiny		22.1	91.6	86.5	93.2	95.1	10.9
YOLOv5s [19]		24.0	93.7	89.2	95.3	96.5	10.0
YOLOv5n		7.2	93.1	88.8	94.6	96.0	14.7
YOLOv7 [20]		127.3	94.2	90.2	96.1	96.4	2.78
YOLOv7-tiny		21.3	93.3	88.4	95.2	96.4	10.3
YOLOv8s		28.7	93.8	89.9	95.6	96.8	8.13
YOLOv8n		8.2	93.0	88.0	94.6	96.5	14.9
Accuracy Priority Mode	640 × 640	17.8	93.8	89.1	95.2	97.1	9.80
Speed Priority Mode		3.4	92.9	88.8	94.2	95.8	20.8

**Table 7:** Experimental results of models with 320 × 320 images.

Model	Input Size	Accuracy (%)				Inference Rate (fps)
		mAP50				
		All	Person	Dog	Cat	
**YOLOv3-tiny**	**320** **×** **320**	90.5	84.8	92.6	94.0	22.2
YOLOv4-tiny		92.4	87.9	94.2	95.2	22.2
YOLOv5s		92.4	87.3	94.2	95.8	29.4
YOLOv5n		91.2	85.1	93.6	94.7	40.0
YOLOv7		93.4	88.4	95.3	96.4	8.33
YOLOv7-tiny		91.7	85.9	93.9	95.2	27.0
YOLOv8s		92.8	87.3	94.9	96.1	22.7
YOLOv8n		90.9	84.7	93.2	94.7	40.0
Accuracy Priority Mode	320 × 320	92.5	87.5	93.9	96.1	22.7
Speed Priority Mode		90.7	85.3	92.9	94.0	50.0

**Table 8:** Comparison of results of MAE value.

Mode	${MAE}_{pixel}$
YOLOv8s	0.223
Accuracy Priority Mode	0.107
Speed Priority Mode	0.103

## Data Availability

The data that support the findings of this study are available from Soonchunhyang University. Data are available from the author with the permission of Soonchunhyang University.
